# Damage and spatiotemporal dynamics of the Ngaio flat mite, *Brevipalpus ferraguti* (Trombidiformes: Tenuipalpidae), with observations on the development of the female insemination system

**DOI:** 10.1007/s10493-021-00670-y

**Published:** 2021-11-05

**Authors:** Hector Alonso Escobar-Garcia, Francisco Ferragut

**Affiliations:** 1 Facultad de Agronomía, Universidad Nacional de Piura (UNP), Urb. Miraflores S/N, Castilla, Piura Peru; 2 Instituto Agroforestal Mediterráneo, Universitat Politècnica de València, Valencia, Spain

**Keywords:** Ornamental pests, Population biology, Seasonal abundance, Aggregation patterns, Spermatheca

## Abstract

We studied the Ngaio flat mite, *Brevipalpus ferraguti* Ochoa & Beard, on *Myoporum laetum* (Scrophulariaceae), a common introduced plant used as hedgerows in gardens and green areas of the Mediterranean, where the mite causes considerable damage. We first describe the damage, and then the patterns of mite seasonal abundance and spatial distribution. Finally, we address the development of the female insemination system at the population level. Damage occurs on both sides of the leaves, starting with a uniform stippling and bronzing and ending in the leaves drying out and extensive defoliation that coincides with summer. Mite population peaked between June and August, maintained moderate levels in autumn and winter and reached its lowest density in early spring. Active motile immatures and eggs were present throughout the year. Females and motile immature forms were more abundant on the abaxial (lower) leaf surface, but eggs were deposited on both surfaces indistinctly, suggesting that females actively move to the adaxial (upper) surface in summer to oviposit. All the developmental stages were aggregated on the leaves throughout the year regardless of their population density. Our study suggests that a binomial or presence-absence sampling, examining only the number of females on the abaxial surface, can accurately estimate the total mite density levels. Only 23.5% of females possessed a fully developed spermatheca, whereas in 76.5% of the cases the seminal receptacle was not present or not developed. Females with a complete spermatheca were less abundant in summer. Average temperatures and host plant species affected the occurrence of this reproductive structure.

## Introduction

Gardens and green areas in our cities constitute anthropogenic urban ecosystems in which ornamental plants are susceptible to insect and mite outbreaks (Raupp et al. 2010). Urban pest management has traditionally relied on chemical applications using broad toxic pesticides. In recent years, however, there has been an evolution towards more sustainable management strategies in European urban landscapes mirroring the change that took place in agricultural ecosystems. Thus, the 2009/128/EC Directive on the Sustainable Use of Pesticides (SUDP) established a reduction in and the restriction of the use of pesticides not only in the production of food products, but also on plants in public parks, gardens and recreation grounds, whereby the use of these chemical substances is to be minimised or totally prohibited (EPC 2009).

One of the most common plants in urban green areas of Mediterranean Spain is ‘*mioporo*’ or ‘*ngaio*’, *Myoporum laetum* G. Forst. (Scrophulariaceae). Native to New Zealand, it was introduced to Spain as an ornamental plant and is commonly used in gardens, streetscapes and even in agricultural areas as hedgerows to indicate boundaries between crops and to provide barriers to the wind. A few years ago, the flat mite *Brevipalpus ferraguti* Ochoa & Beard (Trombidiformes: Tenuipalpidae) was described on *M. laetum* from gardens in the city of Valencia, Spain (Beard et al. 2015). This herbivorous mite is polyphagous, developing on a taxonomically broad range of ornamental plants such as *Pittosporum tobira* (Thunb.) W.T. Aiton (Pittosporaceae), *Tecomaria capensis* (Thunb.) Lindl. (Bignoniaceae), *Eleagnus pungens* Thunb. (Eleagnaceae), *Brugmansia candida* Pers. (Solanaceae) and bitter orange, *Citrus aurantium* L. (Rutaceae) (pers. obs.). Recently, it has been reported from Brazil and Bolivia on various host plants belonging to the families Solanaceae, Oleaceae and Malvaceae (Tassi 2018; Tassi et al. 2018). In Spain, it reaches highest populations on *M. laetum*, where it reduces the plants’ aesthetic quality causing discoloration of the leaves and severe defoliation. To date, application of chemical pesticides is the only effective solution against an infestation caused by this species, or damaged plants have to be substituted with other, more resistant plant species. In order to maintain ornamental variety while also complying with the regulations of the European Union on the use of pesticides, the establishment of integrated pest management (IPM) programs against urban pests is desirable, and the first prerequisite for developing and implementing IPM is a better understanding of the population biology and ecology of the pest and how these relate to the damage caused to the plants.

Taxonomically, *B. ferraguti* belongs to the *phoenicis* species group within the genus *Brevipalpus*, which contains some of the most damaging flat mites (Jeppson et al. 1975; Childers et al. 2003). After the taxonomic revision of the genus by Beard et al. (2015) it became evident that most of the previous reports of the species *Brevipalpus phoenicis* (Geijskes) correspond, in fact, to other species. This means that much of the information published on the biology of *B.* ‘*phoenicis*’ is doubtful or uncertain, as the observations were probably based on incorrect species identifications. The two most extensive studies on the biology, damage and control of *B. phoenicis* produced in the second part of the twentieth century were published by Haramoto (1966) and Oomen (1982). According to recent examinations of type material and voucher specimens deposited in museums and private collections it is probable that the mites studied on papaya in Hawaii by Haramoto were, in fact, *Brevipalpus papayensis* Baker, and the detailed study of *Brevipalpus* on tea plantations from Indonesia corresponds to *B. papayensis* or *Brevipalpus yothersi* Baker (both assumptions based on the information provided by Beard et al. 2015). Herein, we will use the specific name *phoenicis* between quotation marks when the species identity most probably refers to another taxon.

But the interest of *Brevipalpus* mites goes beyond just their economic importance as crop or ornamental pests. The genetic and reproductive biology of these species is astonishing. Weeks et al. (2001) demonstrated that the females of the species then considered *B. phoenicis* are haploid. One of the most controversial issues is the presence of a female insemination system or spermatheca in thelytokous species with the complete absence (or very reduced numbers) of males. Moreover, it has been observed that in some *Brevipalpus* species, a proportion of the females in a given population has a complete spermatheca with a developed seminal receptacle, whereas other females in the same population lack a developed seminal receptacle and the insemination duct merely terminates in a small and non-sclerotized vesicle (Beard et al. 2015; Di Palma et al. 2020). The reasons for this intra-populational variability and its implications in the population biology of the species are still unknown.

In this study, we describe the damage caused by *B. ferraguti* on *M. laetum* for the first time. Next, we analyse the seasonal variation in abundance and the spatial distribution of *B. ferraguti* on the plant, to provide the information necessary for developing future sampling plans. In addition, we investigate whether the presence of a fully developed spermatheca (insemination duct plus seminal receptacle) is related to the season of the year, average temperatures or species of host plants.

## Material and methods

### Study site and climatic characteristics

Monitoring was conducted in the Viveros Garden located in the city of Valencia, Spain (39° 28′ 39″ N, 0° 22′ 06″ W, 12 m above sea level). The garden covers a surface area of about 19 ha and includes many pure hedgerows of *M. laetum*. Moreover, the site is the type locality for *B. ferraguti*; that is, the exact place where the mite specimens used for the original description of the species were collected.

The city of Valencia presents a typical Mediterranean coastal climate, with mild winter temperatures seldom below 5 °C, and dry and hot summers. During the period of monitoring (November 2014–November 2015), rainfall reached an annual value of 493.6 mm and rain periods were concentrated during April–May and October. The climatic data used for this study (average daily temperatures and relative humidity for the sampling period) were obtained from the Viveros Weather Station (AEMET 2020) located within the garden, about 300 m from the sampled plants (39° 28′ 50″ N, 0° 21′ 59″ W, 11 m a.s.l.).

### Sampling procedures and analytical approaches

We collected samples from unsprayed plants every 2 weeks from November 2014 to November 2015. Firstly, we studied the changes in mite abundance on *M. laetum* throughout the year. On each date, 50 fully developed leaves were randomly collected. Leaves were put in paper bags, transported to the laboratory inside a portable cooler and examined under a stereo microscope. We recorded the number of mites, separating the values for eggs, motile immatures (larvae + protonymphs + deutonymphs) and adult females (the number of males was negligible). Next, we determined the effect of the season, temperature and plant phenology on mite density. As values for mite abundance were not normally distributed and variances were not homogeneous, we performed the non-parametric Kruskal–Wallis H test to compare the population density (average number of mites per leaf) for total mites and each developmental stage, throughout the seasons of the year, followed by post hoc multiple comparison test (α = 0.05). Furthermore, on each sampling date we checked the plant phenology in order to detect both the onset and the duration of the main physiological events that occur throughout development (e.g., new leaf sprouting, blooming).

Secondly, we used the same samples collected to determine the mite densities and their location on adaxial (upper) or abaxial (lower) surface of the leaf. We performed Wilcoxon rank tests in order to establish intra-leaf distribution patterns for each developmental stage, and to test whether they showed preferences for living on the upper or lower leaf surfaces.

Thirdly, we studied the aggregation behaviour of mites at leaf level using Taylor’s power law as a prerequisite to develop further sampling plans. This power law describes the relationship between the mean density and the variance of all samples collected using the equation *s*^2^ = *am*^*b*^; where *s*^2^ is the sample variance, *m* the sample mean density, and *a* and *b* are Taylor’s coefficients (Taylor 1961). Coefficient *a* is related to the nature and scale of the sampling scheme, and is affected by the sampling size, whereas coefficient *b* describes an intrinsic property of the spatial distribution of the population in a particular environment. Coefficient *b* is considered the aggregation index, and values higher than 1.0 describe an aggregated population (Taylor 1984). Taylor’s coefficients were calculated by means of a simple regression analysis after applying logarithms to the density and variance values (after log_10_[x + 1] transformation) for each sampling date (log *s*^2^ = log *a* + *b* log *m*). Multiple regressions were calculated: the total mite population on both adaxial and the abaxial leaf surfaces; the same for total egg numbers, motile immatures and females on the whole leaf, and for both the adaxial and abaxial surfaces. The model’s overall goodness of fit was determined by estimating the values of *r*^2^. Two-tailed t-tests (*df* = n − 2, α = 0.05) were used to determine whether the slopes of the regression lines were significantly different from 1.0. Values of *b* were compared using 95% confidence intervals.

### Factors affecting development of female spermatheca

We examined the morphological structure of the female insemination system to find a relationship between its state of development (undeveloped without seminal receptacle or developed with full seminal receptacle) and the (1) season of the year, (2) climatic variables, and (3) host plant. Females sampled from *M. laetum* were collected from the same leaves used to assess the population density. In order to determine the effect of host plant, we also collected *B. ferraguti* from two other common ornamental plants in the Viveros garden in which the mite also occurs, *P. tobira* and *T. capensis*. As *Brevipalpus californicus* (Banks) species B (according to the provisional classification of the *californicus* species group by Beard et al. 2012) was also collected from *P. tobira*, we examined their spermathecae as part of the study. Ten branches, each composed of 8–12 leaves of *Pittosporum* and *Tecomaria* were taken on each date. In all cases, 40 adult females were randomly collected on each sampling date per plant species; for the instances in which the sample size was < 40, all available females were analysed. Females were partially clarified in Nesbitt fluid, mounted with Heinze-PVA medium (Krantz and Walter 2009) and stored in an oven at 50 °C until fully clarified. Females were later examined at 400 × and 1000 × magnification using differential interference contrast (DIC) on an Eclipse Ni-U compound microscope (Nikon, Tokyo, Japan). Microscopic images included in the publication were captured with a Nikon DMX 1200 digital camera. Voucher specimens were deposited in the Acarology Laboratory of the Mediterranean Agroforestry Institute (IAM), of the Universitat Politècnica de València (UPV).

Kruskal–Wallis non-parametric tests were performed to assess whether the season of the year affects the proportion of females with or without developed spermathecae on *Myoporum* leaves, and to compare the presence of complete or incomplete spermathecae among plant species. The effect of average temperatures on the development of spermathecae was tested by Spearman's rank correlation coefficient. For this analysis, the mean, maximum and minimum temperatures were obtained from the period between two consecutive sampling dates.

All statistical analyses were performed using Centurion v.18 (Statgraphics 2009) and IBM-SPSS v.19 (SPSS 2011) software, with α = 0.05.

## Results

### Mite external morphology and characterization of damage on *Myoporum laetum*

**Fig. 1:**
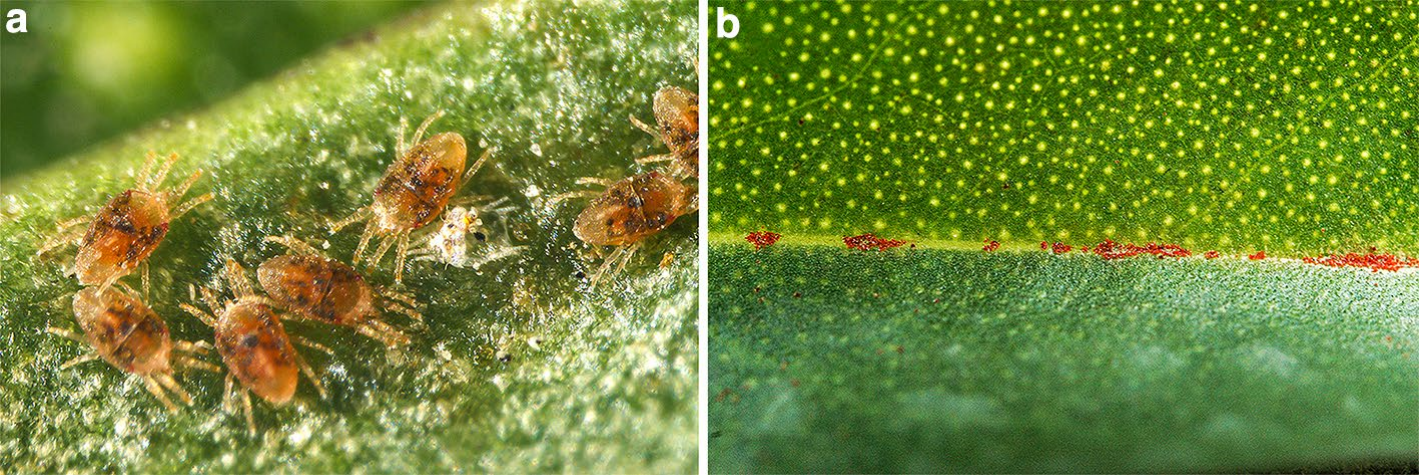
**a** Females of *Brevipalpus ferraguti* on *Myoporum laetum*. **b** Clusters of eggs on the adaxial midrib

Colonies of the Ngaio flat mite (NFM) occur on leaves, buds, and green twigs of the host plant, but were not observed on fruits. The females exhibit the general morphology common to all *Brevipalpus* species, being orange-red and with several irregular dark spots located on the dorsolateral region and along the sejugal groove (Fig. 1a). The male is smaller than the female and has a more flattened body, and the immature stages are similar to the adults in colour. The eggs are elliptical in shape, bright red and females deposit them often in large numbers, along the midrib, and inside depressions or damaged areas on both sides of the leaves, where mites tend to aggregate. The brightly red egg masses on the midribs are visible to the naked eye (Fig. 1b). Adult females of the NFM are more robust and distinctly broader than *B. californicus*, with which they often co-exist.

**Fig. 2:**
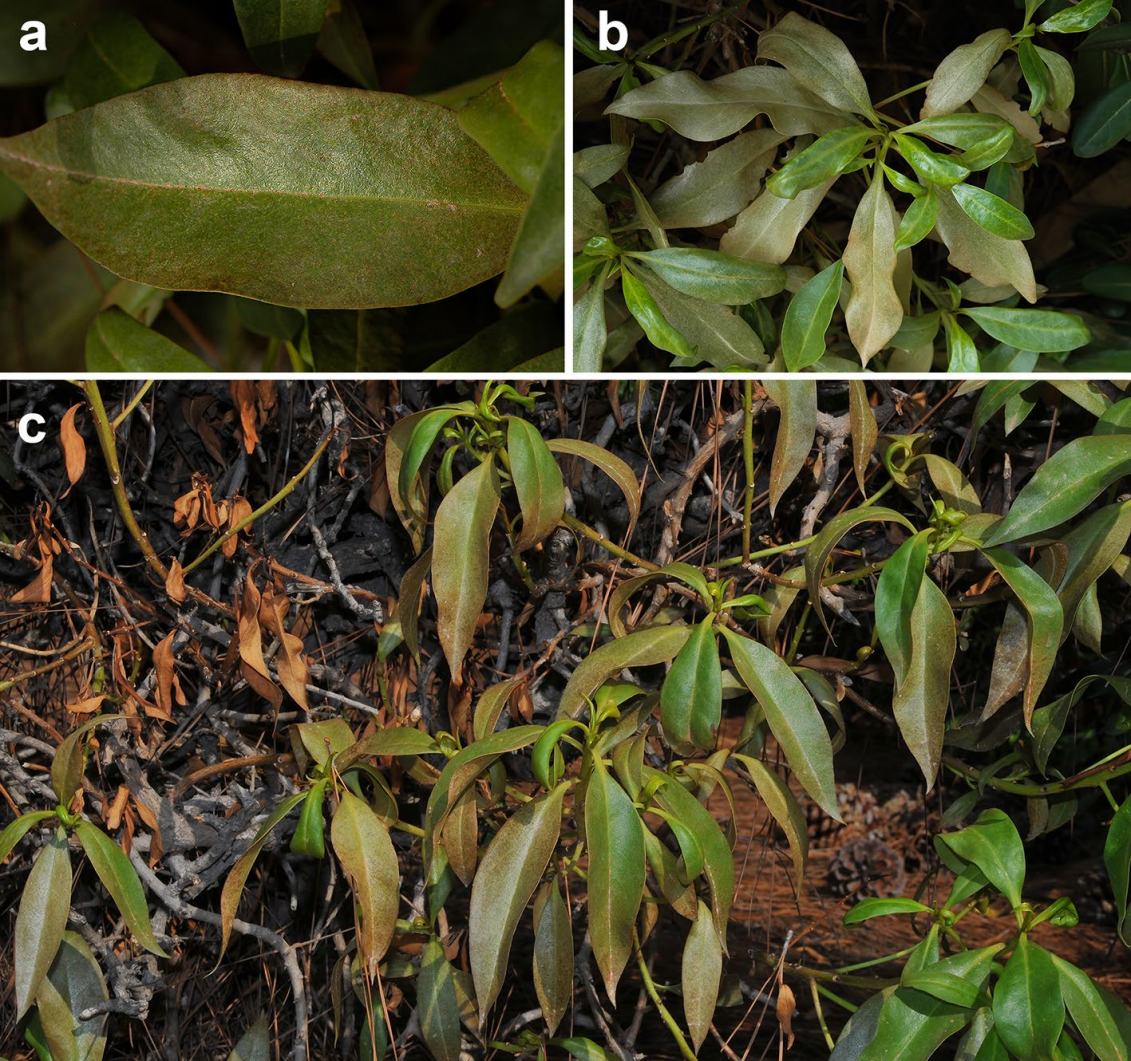
Damage caused by *Brevipalpus ferraguti* on *Myoporum laetum.* **a** Initial damage on leaves. **b** Seriously affected, whitish leaves in comparison to new, green leaves. **c** Strongly affected plants at the end of summer showing brownish discoloured leaves, completely dry leaves, and intense defoliation

**Fig. 3:**
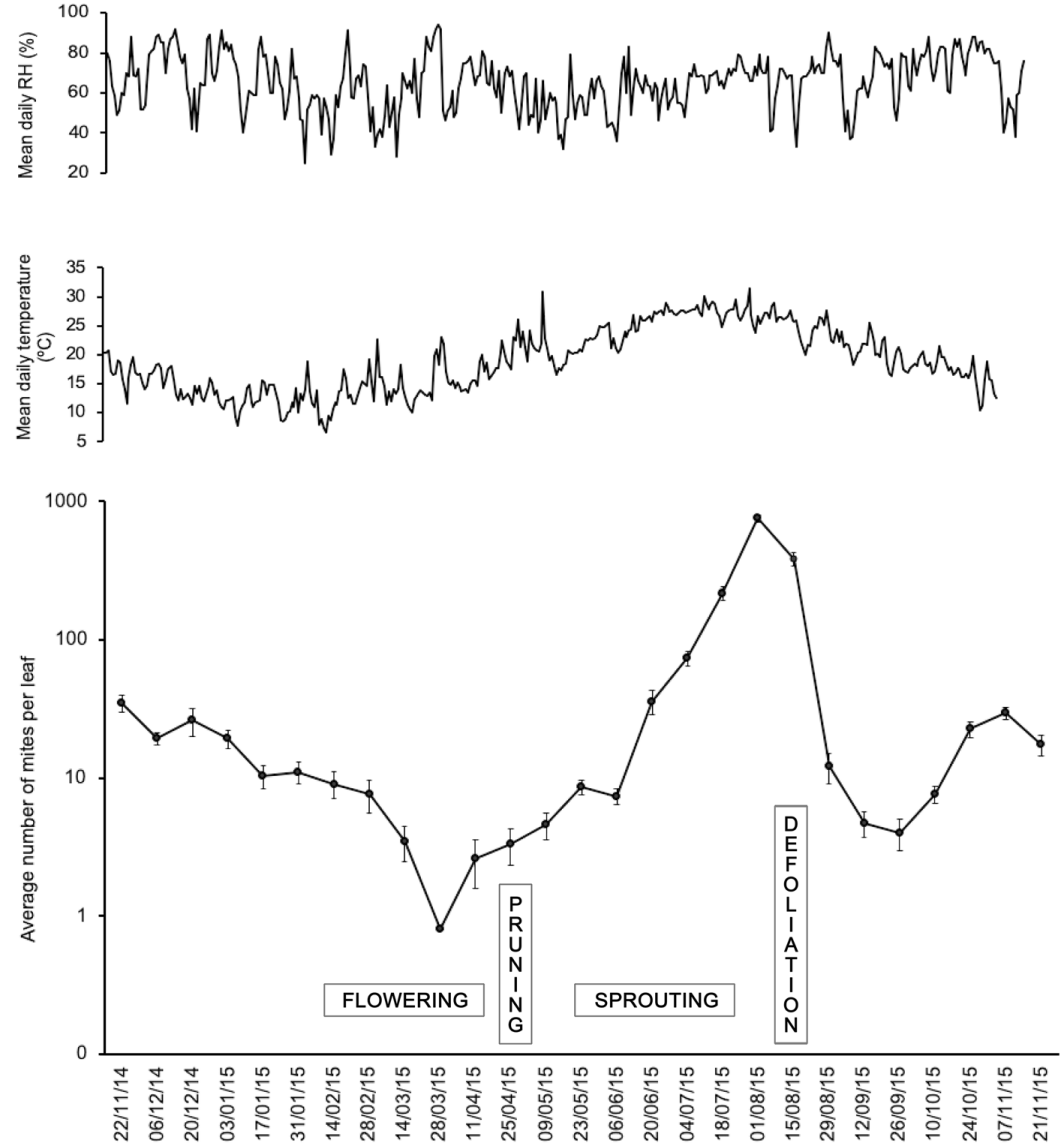
Average annual distribution of *Brevipalpus ferraguti* density on *Myoporum laetum* (mean ± SE number of mites per leaf). Values plotted on a logarithmic scale. The top two panels show the mean daily temperature and relative humidity (RH). Plant phenology periods and other events are indicated on the graph

The type of damage produced depends on the population levels, with symptoms ranging from stippling or silvering of the leaves to death of leaves and stems (Fig. 2a–c). After leaf colonization, feeding injury results in a uniform stippling that later becomes bronze-coloured. As the mite population increases, the damage extends to both sides of the leaves and can affect the entire surface area. At that moment, marginal necrosis can be observed on many leaves (Fig. 2a). At this point, if the plant sprouts, young and green leaves stand out from the old, dry, and whitish leaves, where most of the mites still remain (Fig. 2b). Higher infestations occur in summer and frequently heavy defoliations are observed in some plants by the end of summer. At that time, new and still green leaves coexist with discoloured, brown, dry leaves, and partially defoliated stems (Fig. 3c). All the symptoms observed were consistent with the elimination of epidermic and parenchymal cells and the subsequent consequences of this, such as leaf discoloration, drying, and defoliation. No symptoms consistent with virus transmission were observed on *Myoporum*, *P. tobira* or *T. capensis*.

### Seasonal trends and population structure

NFM showed only one period of strong population increase during the year of study, over summer (Fig. 3). Mite abundance started to increase in June, and reached the highest value of 757 mites/leaf by early August. After that, the density rapidly declined, reaching values of only 4 mites/leaf by the end of September, but rose and fell slightly over autumn and winter, with densities between 8 and 35 mites/leaf. The population then declined again at the beginning of spring, reaching the lowest values of the year with an average of only 0.8 mites/leaf. Mites were overall more abundant in summer than in all the other seasons (*H* = 9.861, *df* = 3, *p* = 0.019), and were more abundant in autumn than in winter and spring.

We observed synchrony between mite abundance and plant phenology (Fig. 3). The population growth at the beginning of summer coincided with the sprouting period. Likewise, the reduction of the mites in spring appeared to be associated with the flowering period. Finally, the sudden population drop after the summer peak coincided with strong defoliation in many plants.

**Fig. 4:**
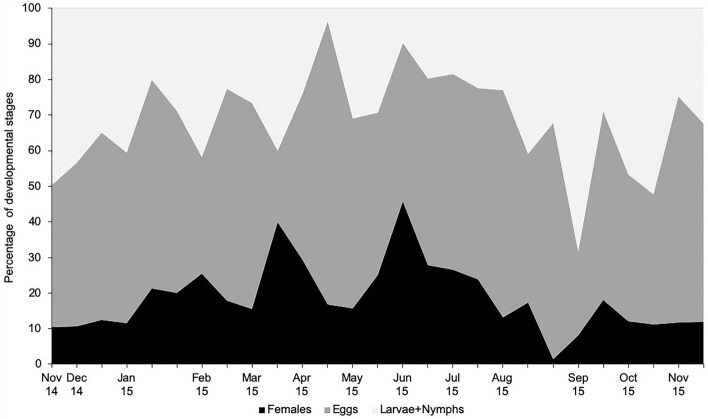
Development in age structure of *Brevipalpus ferraguti* females, eggs and larvae + nymphs, from November 2014 to November 2015 on *Myoporum laetum*

Active motile immatures and eggs were present throughout the year, even in the coldest periods, demonstrating that the mite did not undergo diapause. Immatures were more abundant than adults in all the seasons, ranging from 55 to almost 100% of individuals depending on the period of the year (Fig. 4). Maximum values of immatures were observed in late summer and autumn, and in the latter season immatures represented about 90% of all individuals. The proportion of females in the population showed two distinct peaks, in early spring and early summer, and they account for 40–45% of the total population, respectively. In general, an increase in the percentage of females was followed by an increase in eggs and subsequently of motile immatures. Females were not significantly more abundant in summer than in other seasons (*H* = 2.19, *df* = 3, *p* = 0.53), and we found no differences among the remaining seasons. On the contrary, eggs and motile immatures (larvae + nymphs) were significantly more abundant in summer (eggs: *H* = 7.665, *df* = 3, *p* = 0.043; remaining immatures: *H* = 14.146, *df* = 3, *p* = 0.003) than in the other seasons of the year. In this study, males represented only 0.8% of adults on *Myoporum* (total adults n = 898), 1.2% on *Pittosporum* (n = 174) and 0.6% on *Tecomaria* (n = 532). The total percentage of males collected from all adults combined was 0.8% (n = 1604).

### Intra-leaf distribution patterns and aggregation behaviour

**Fig. 5:**
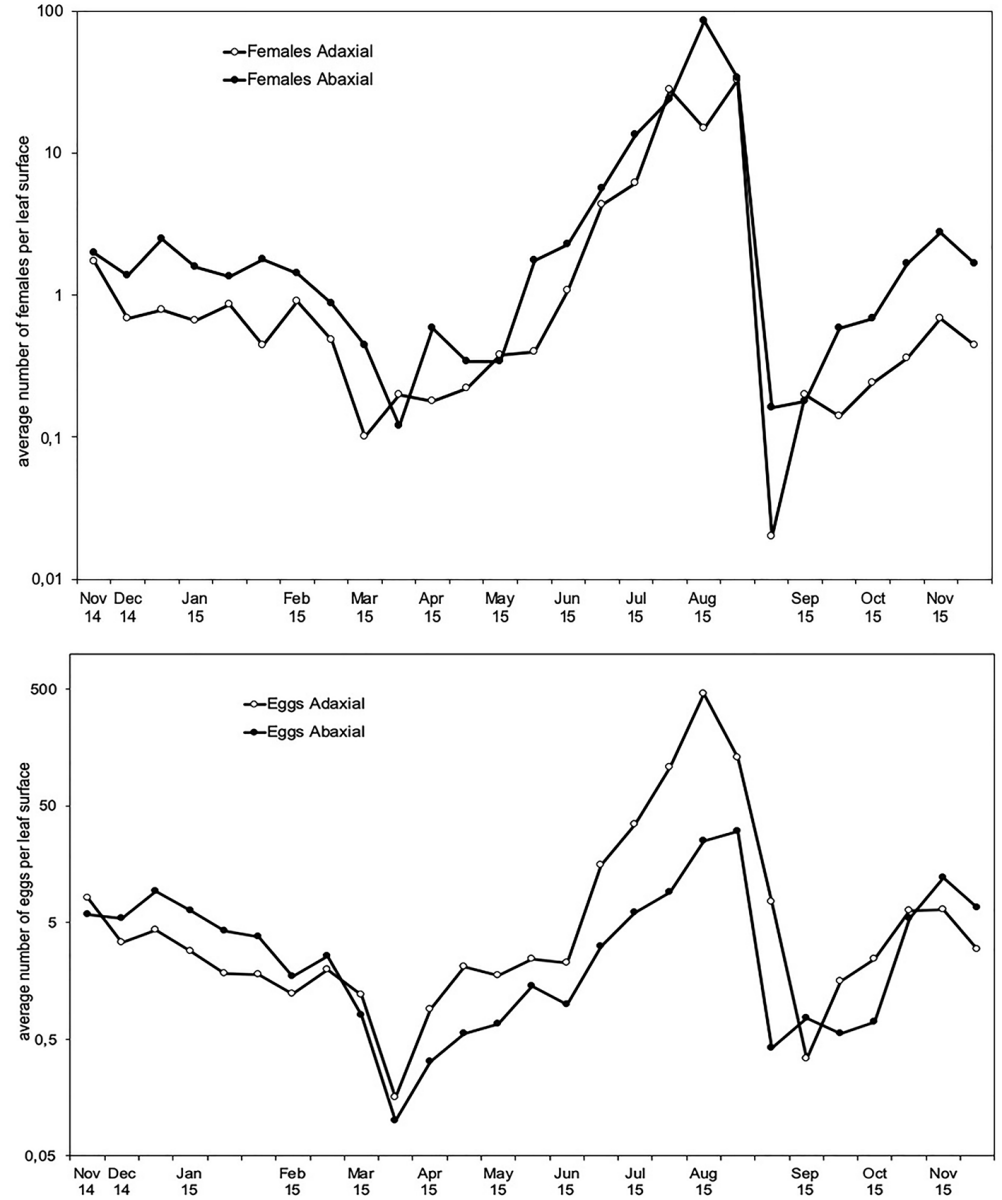
Density of *Brevipalpus ferraguti* females and eggs throughout the year on the upper (adaxial) and lower (abaxial) leaf surfaces (mean number per leaf surface). Note that the scale on the y axes is logarithmic and different between the panels

Although the motile stages of NFM exploited both leaf surfaces, they each had different preferences. When we consider the whole year, females and immature motile forms preferred to live on the abaxial (lower) surface (Wilcoxon test, females: *p* = 0.00015; motile immatures: *p* = 0.00007), but females deposited their eggs equally on both surfaces without showing a preference. The distribution on the upper and lower surfaces showed a similar trend throughout the year for all stages, with densities peaking in summer. However, seasonal changes in the spatial distribution were observed in the case of eggs (Fig. 5). During most of the year all the stages (including eggs) were more abundant on the lower side of the leaves, but the eggs were more abundant on the upper surface in summer. During the summer, the upper (adaxial) leaf surface had 10 × more eggs than the lower surface, compared to only 1.3 × during the rest of the year. Concurrently, the density of females on the lower surface experienced a reduction during summer, with 1.9 × more females on the lower surface than the upper surface compared to 2.4 × in the rest of the year (although statistical significance was not achieved) (Fig. 5). We applied regressions to reveal whether the preference of females to lay eggs on the upper leaf surface was affected by the density of other developmental stages. This preference was neither influenced by egg density (r^2^ = 0.13), female density (r^2^ = 0.14) nor motile immature density (r^2^ = 0.16).

The application of Taylor’s power law provided a good fit to the data for comparisons of the life stages between entire leaves, and the life stages on adaxial versus abaxial leaf surfaces. Coefficients *b* of Taylor were always significantly > 1, indicating that all the sampled forms tended to aggregate on the leaves (Table 1). If we consider the complete leaf, the aggregation of females, motile immatures, and eggs did not differ among them, with *b* coefficients ranging between 1.43 and 1.45. Because of the absence of differences, we pooled the data of all the developmental stages resulting in an aggregation coefficient *b* of 1.42.

**Table 1:** Taylor’s power law indices for all developmental stages, females, motile immatures and eggs of *Brevipalpus ferraguti* on complete leaves and on adaxial and abaxial leaf surfaces (n = 27)

Life stage	*a*	*b*	95% confidence interval (*b*)	r^2^	t^a^
All forms on complete leaves	10.54	1.42	1.39–1.44	0.964	7.73
Females on complete leaves	0.93	1.45	1.43–1.47	0.971	9.11
Larvae + nymphs on complete leaves	6.51	1.43	1.40–1.45	0.957	7.06
Eggs on complete leaves	10.42	1.45	1.42–1.46	0.966	8.21
All forms on adaxial surface	10.01	1.44	1.41–1.46	0.956	7.22
All forms on abaxial surface	9.25	1.36	1.33–1.38	0.954	6.04
Females on adaxial surface	0.98	1.38	1.36–1.40	0.963	7.13
Females on abaxial surface	1.11	1.42	1.40–1.44	0.958	7.15
Larvae + nymphs on adaxial surface	4.17	1.45	1.42–1.47	0.978	8.04
Larvae + nymphs on abaxial surface	6.32	1.38	1.36–1.41	0.947	5.92
Eggs on adaxial surface	12.31	1.43	1.40–1.45	0.958	7.21
Eggs on abaxial surface	11.39	1.35	1.31–1.40	0.852	3.16

^a^t-value for slope = 1

On the contrary, significant differences in the aggregation pattern between the upper and lower leaf surfaces were observed for all the developmental forms, although the preference for either surface was different depending on the life stage. Females congregated mainly on the lower surface (*b* = 1.42 versus *b* = 1.38 for the upper), whereas eggs and larvae + nymphs were more clumped on the upper surface. Values of *b* for the latter stages were significantly higher on the upper surface, with *b* ranging between 1.43 and 1.45 in eggs and motile immatures, respectively.

### Factors affecting the development of spermatheca

**Fig. 6:**
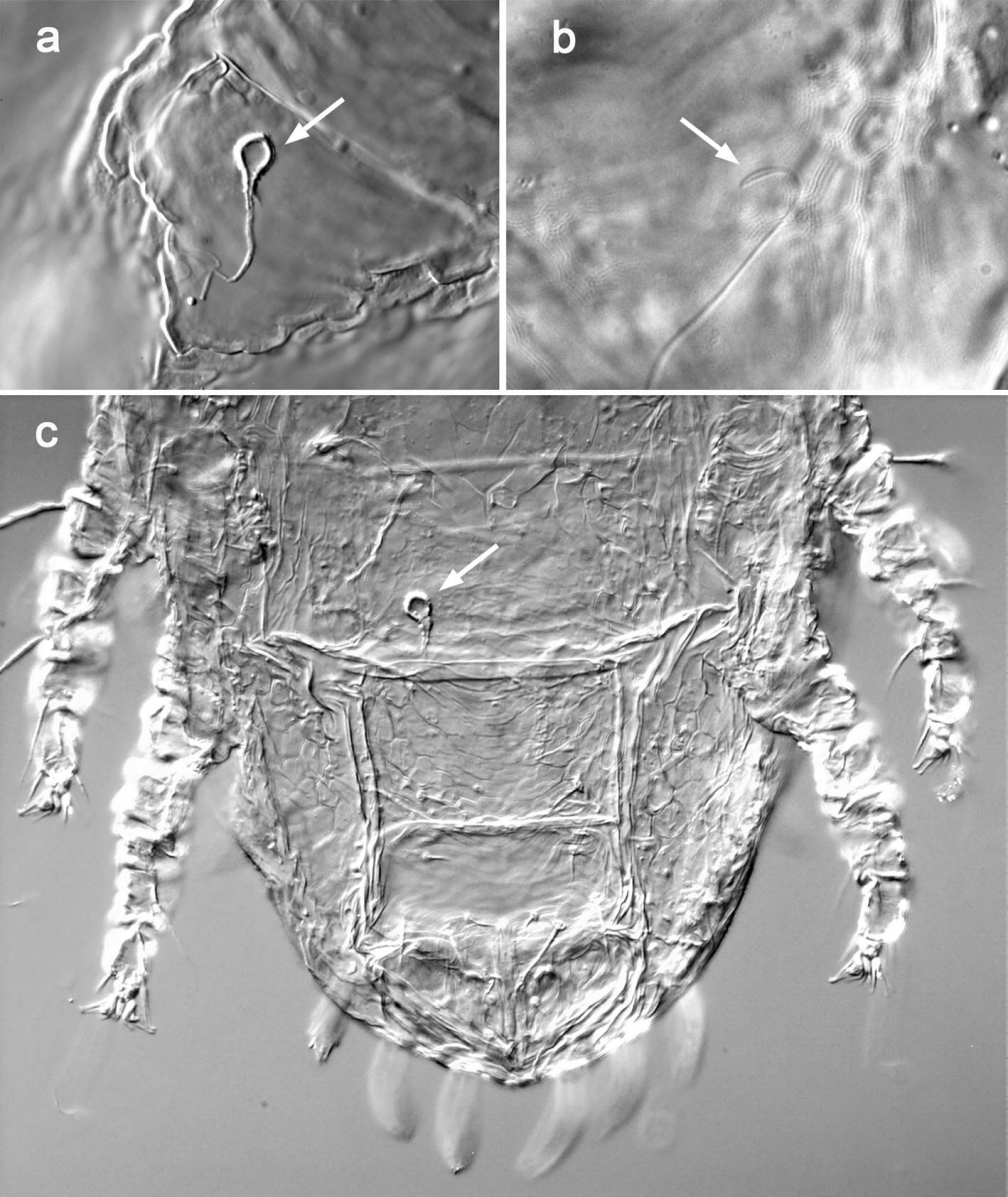
Development of the seminal receptacle or spermatheca in *Brevipalpus ferraguti*. **a** Fully developed seminal receptacle. **b** Undeveloped spermatheca. **c** Pharate female showing the complete spermatheca. The deutonymphal condition can be ascertained by the presence of the long dorsocaudal setae *e3*, *f3*, *h2*, *h1*

**Fig. 7:**
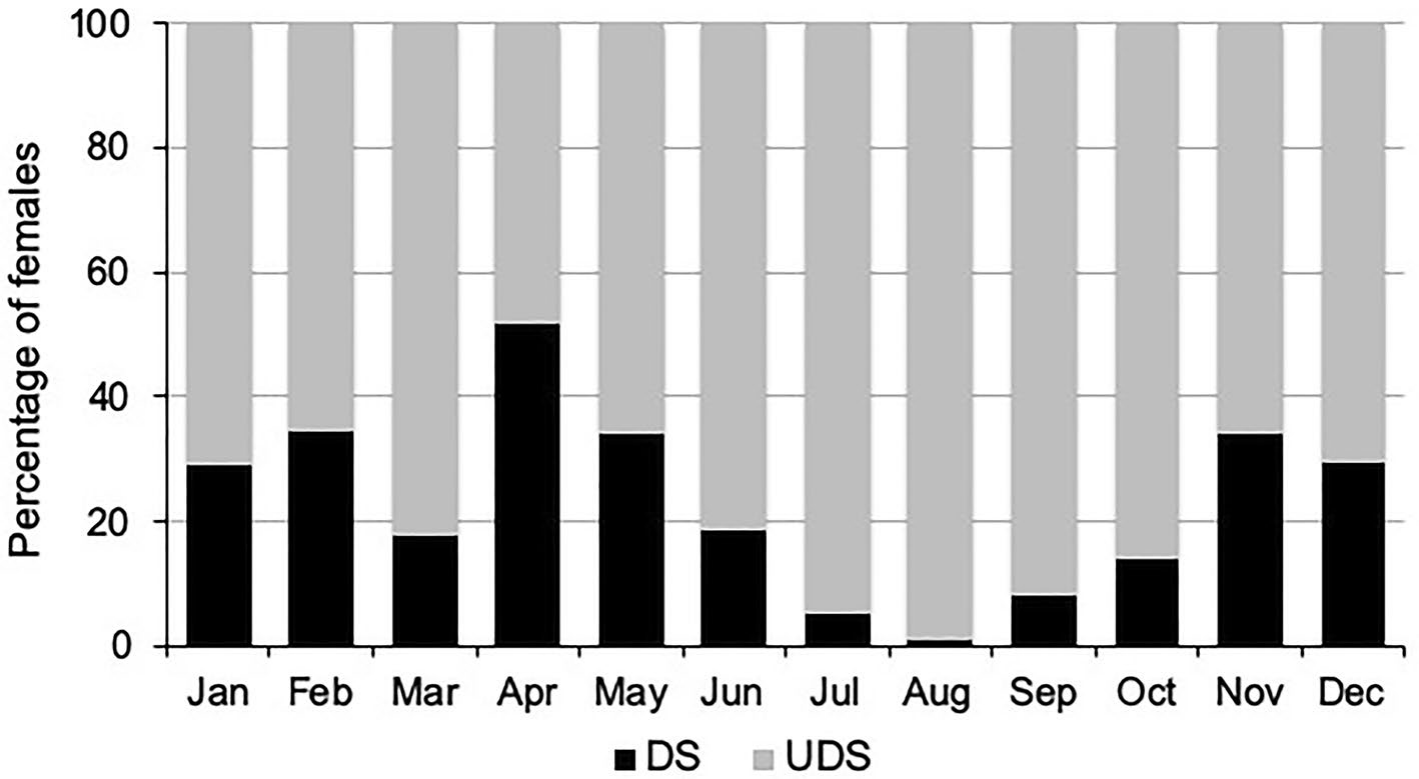
Monthly distribution (%) of females of *Brevipalpus ferraguti* on *Myoporum laetum* with fully developed spermatheca (DS) or undeveloped spermatheca (UDS) (total n = 891)

The insemination system of 891 females collected throughout the year on *M. laetum* was examined in order to detect the presence (fully developed spermatheca) or absence (undeveloped spermatheca) of the seminal receptacle. In 209 cases (23.5%), the spermatheca clearly showed a well-developed, well-sclerotized, and specifically shaped seminal receptacle (Fig. 6a); whereas in 682 cases (76.5%), the seminal duct terminated in a small, poorly sclerotized and bulbous expansion (Fig. 6b). The proportion of females in the population with a complete spermatheca was affected by the time of year, with much lower numbers in July–September (Fig. 7), and significantly more undeveloped spermathecae in the summer (*H* = 13.08, *df* = 3, *p* = 0.004). The occurrence of complete spermathecae was associated with the average maximum and minimum temperatures, the proportion of females with a full spermatheca decreasing with increasing mean temperatures (Spearman's rank correlation: r =  − 0.589, p = 0.0027; n = 26).

In total 1592 females, collected from three plant species—*M. laetum*, *P. tobira* and *T. capensis*—were examined, from which 280 (17.5%) had developed a spermatheca and 1312 (82.4%) lacked any seminal receptacle. The presence of complete spermathecae was greater in females developing on *M. laetum* than in females on the other host plants (*H* = 34.49, *df* = 2, *p* = 0.032); the occurrence (%) of spermathecae in females collected on both *P. tobira* and *T. capensis* was the same, regardless of the time of year.

We noted that the proportion of females with developed spermathecae may also differ among *Brevipalpus* species. We checked the insemination system of 461 females of *B. californicus* species B feeding on *P. tobira*, and found that 402 (87.2%) showed a complete spermatheca and the remaining 59 (12.8%) lacked any seminal receptacle. We did not find males of this species during all the sampling period.

## Discussion

### Population biology of the Ngaio flat mite

Since its original description in 2015 (Beard et al. 2015), a lot more is now known about the biology of the NFM. The mite has a much wider geographical range than originally described, as it has recently been reported from Brazil and Bolivia, based on morphological and molecular evidence (Tassi 2018; Tassi et al. 2018). Likewise, it appears to be more polyphagous than first reported as it has been found from hosts belonging to different plant families, where feeding, development and reproduction were confirmed by the occurrence of eggs and all the motile forms (Tassi et al. 2018; this study). Our observations of the symptoms produced by feeding indicate that NFM causes stippling and browning of host leaves and the feeding can result in extensive defoliation. According to the literature, similar symptoms to those reported here were observed for *B*. ‘*phoenicis*’ on various crops and non-cultivated plants around the world [see Ochoa et al. (1994) and Childers et al. (2003) for details of symptoms caused by *B. phoenicis* sensu lato (s.l.) on different ornamental and cultivated plants]. Importantly in Brazil, NFM has been collected on the plant genera *Cestrum* and *Solanum* (Solanaceae) infected by ringspot viruses (CnRSV, SvRSV), and on *Ligustrum* (Oleaceae) affected by *Ligustrum leprosis virus* (LigLV) (Tassi et al. 2018), and consequently is implicated in the transmission of these viruses.

Weather conditions in the studied area were favourable for continuous reproduction and development throughout the year, and there was no indication of any diapausing behaviour taking place. Nevertheless, the fluctuations in the number of individuals were extreme when different periods of the year were compared. The mite showed an annual peak produced by exponential growth between early June and the end of July. The existence of just one period of intense population growth agrees with that reported for *B*. ‘*phoenicis*’ by Haramoto (1966), Oomen (1982) and Mineiro et al. (2008). The latter study probably refers to either *B. papayensis* or *B. phoenicis* feeding on coffee plants that were affected by a ringspot disease in São Paulo State, Brazil (according to the data provided by Tassi 2018).

These fluctuations in mite abundance might be promoted by climatic conditions, plant phenology, plant defence responses, or a combination of all these factors. The onset of the population peak in June was coincident with the sprouting period, when new leaves with highly nutritive cell content are developing. During the approximately 7-week period of continuous population growth, average temperatures increased 7.2 °C (from 20.9 °C on June 6th to 28.1 °C on August 1st). Consequently, mite density increased > 100 × , from 7.4 to 757 mites/leaf. At the start of August, the leaves that supported such a high number of mites became depauperate and dry and eventually began to fall for about 5 weeks, from mid-August to the end of September. At this point, the population drop was rapid, reducing > 96 × , from 384.8 to 4 mites/leaf, even though mean temperatures remained fairly high, diminishing only 3.5 °C (from 27.5 to 24 °C). Drying of the leaves was probably accentuated by the absence of rain during summer, and the consequent combination of mite infestation and drought stress promoted the fall of leaves. Another underlying cause of the density reduction was probably the reduced mobility of the mite. It is likely that most of the individuals remained on the old and dried leaves when they dropped and only a small part of the population was able to disperse to the new leaves. This apparently limited ability to disperse was also demonstrated by Haramoto (1966) for *B*. ‘*phoenicis*’. Haramoto suggested that the mites tended to remain in the original area where they were born, and states that the use of wind was the most important method of dispersal for these mites as crawling from plant to plant is only possible in areas of closely set plants.

Striking in the annual changes of abundance was the population decline observed in March when spring is coming, as temperatures slowly begin to increase again and plants are entering the flowering period. It is well known that host plant phenology may strongly influence the performance of phytophagous mites, and through the host plant quality to play a major role in shaping their population dynamics (Awmack and Leather 2002). Developing flowers and fruits demand nutrients (mainly sugar and amino acids) which are redirected away from leaves, causing a considerable decline of vegetative growth and the availability of nutrients for leaf-feeding mites (Legaz et al. 1982; Lea-Cox and Syvertsen 1996). An alternative explanation for the mite population decline may be the production of toxic compounds by the plant. The sesquiterpene ngaione is a major secondary metabolite in *M. laetum* that constitutes 70–80% of essential oils and is toxic for animals including livestock (Fuller and McClintock 1986) (the numerous glands containing essential oils can be seen in Fig. 1b). Seasonal variations in ngaione content in leaves were described by Mohamed and Omer (2009), who obtained maximum concentrations in February matching the period in which the mite abundance begins to collapse. It has been demonstrated that carbohydrates and proteins are feeding stimulators for herbivorous mites whereas plant secondary metabolites related to defence, such as phenolic compounds, act as feeding inhibitors (Petanović and Kielkiewicz 2010). More precisely, the increase of the ratio between plant defence compounds and carbohydrates/proteins limits the infestations of the blackberry eriophyid mite *Epitrimerus gibbosus* (Nalepa) (Shi and Tomczyk 2001). Whatever may have caused the population drop, mite density was reduced 9.5 × in about 4 weeks (from 7.6 to 0.8 mites/leaf) although temperature was about stable.

### Spatial distribution

Although all the developmental stages of NFM exploit both upper and lower surfaces of the host leaves, a larger proportion of motile forms (females, nymphs and larvae) was present on the lower surface, and eggs were placed on both surfaces without distinction. This behaviour suggests that a part of the females move to the upper leaf surface in order to deposit eggs, and that after hatching the larvae and nymphs actively move to the lower surface. When regressions were performed, we found that the tendency of females for depositing the eggs on the upper surface was independent of egg density, female density and motile immature density. Eggs were preferably laid along the midrib on both leaf surfaces, in crevices or even inside the exuvia of mites or small insects (Fig. 1b). Since clusters of eggs are abundant and can frequently be found, this may indicate that females return to the same place to deposit their successive eggs. A similar egg deposition behaviour was mentioned in *Brevipalpus* s.l. on several ornamental and cultivated plants, such as *Anthurium andraeanum* (Araceae), *Chamaedorea* spp. (Arecaceae) and the rough lemon *Citrus jambhiri* (Rutaceae) (Ochoa et al. 1994). In a study of intra-leaf preference for several mite families on *Viburnum erosum* (Adoxaceae) and other trees and shrubs in Japan, Sudo and Osakabe (2011) found that the ratio of *Brevipalpus obovatus* Donnadieu eggs collected from adaxial leaf surfaces versus abaxial surfaces was significantly higher than the ratio for the motile stages. They argued that oviposition on the upper leaf surface might provide a benefit for *B. obovatus* as predatory mites like phytoseiids prefer to live on the lower surface, and may decrease predation risk (if the eggs are invulnerable to solar UVB radiation). When movement and selection are feasible and possible, NFM prefers to live on the underside of leaves. This preference may be because the leaf underside offers more protection against environmental factors, provides higher humidity, or perhaps epidermic and parenchymal cells on the lower leaf surface are more nutritious and/or palatable (or easier to access) for the mites.

### Sex-ratio and development of female spermathecal system

According to our findings, NFM exhibits spanandry, that is, the occurrence of very few males in a thelytokous species (Helle et al. 1980). Spanandry appears to be the rule in some of the species related to the NFM and it has been considered the result of inefficient transmission of the feminizing endosymbiont *Cardinium* (Groot et al. 2005). Razoux-Schultz (1961) reported that 1.5% of the population of *B.* ‘*phoenicis*’ on tea in Indonesia was male and, likewise, Haramoto (1966) reported that males represent < 1% of the population on papaya in Hawaii (USA). The role of males in *Brevipalpus* species with thelytokous parthenogenesis has been the subject of some debate. It has been suggested that either the reduced proportion of males are able to copulate but unable to transfer sperm efficiently (W. Helle pers. comm. in Oomen 1982) or that they are incapable of producing sperm at all and are non-functional from the reproductive point of view (Groot et al. 2005). Due to the reduced number of males collected, there were not enough data to correlate their occurrence with any season of the year, with the population growth or with the presence of a fully developed spermatheca in females.

Moreover, the presence of a spermathecal apparatus in females of populations with rare and non-functional males is in itself intriguing. Besides this apparent paradox, the reason why a proportion of the females have a fully developed spermatheca while others in the same population do not, all experiencing the same environmental influences and on the same host plant, still remains unclear. The female insemination system in *Brevipalpus* is composed of a long and thin insemination duct terminating in a seminal receptacle, also called spermatheca (Alberti et al. 2014; Di Palma et al. 2020). Di Palma et al. (2020) investigated its ultrastructural morphology in five *Brevipalpus* species (*B. californicus*, *B. obovatus*, *B. papayensis*, *Brevipalpus tuberellus* De Leon, and *B. yothersi*). They confirmed that the shape of the seminal receptacle is species-specific, stressing thus its importance as a key feature in *Brevipalpus* taxonomy and systematics, as it was previously suggested (Beard et al. 2012, 2015; Navia et al. 2013; Alves et al. 2019), and that females belonging to the same species and population may exhibit developed or undeveloped seminal receptacles. They also wondered whether those differences in the level of development can be attributed to environmental factors, physiological conditions or the age of females. We demonstrated that the development of the seminal receptacle in NFM females was likely affected by climatic variables and average daily temperatures, with fewer being developed in summer. The occurrence of this structure was also negatively correlated with the total population density (r^2^ = 0.42) and with female density (r^2^ = 0.49). There were also differences according to the host plant, with comparatively more developed spermathecae on *M. laetum* than on other hosts. Furthermore, it was evident that the tendency to develop complete spermathecae differed between *B. ferraguti* and *B. californicus* species B, the former expressing mostly undeveloped spermathecae, and the later fully developed spermathecae and reproducing by thelytokous parthenogenesis in our conditions. Although we did not study the influence of female age on the development of the insemination system, we hypothesize that the development of the seminal receptacle is age-independent, as we observed fully developed spermathecae in pharate females, i.e., just before the moult to the adult stage when the adult female can be seen within the deutonymphal integument (Fig. 6c). It would be interesting to examine the state of the spermathecal apparatus of females of known ages, at various times of the year (different seasons), and/or at different stages of the host plant development/biological status. Here we provide quantitative evidence of the variability in the expression of this structure, but the biological significance or underlying mechanisms of its maintenance in females with thelytokous reproduction still remains a mystery.

### Future sampling and decision-making programs

One limitation of our work was that mite populations were monitored for only 1 year. Nevertheless, we can use these limited population behaviour data to develop future sampling designs and pest control decision making. The variance/mean ratio of individual samples was always > 1, demonstrating that the mites were spatially distributed in an aggregated pattern throughout the year regardless of their population density. The Taylor’s coefficients obtained allow the calculation of the number of leaves required to sample the population density with a pre-fixed precision level (Wilson and Room 1983). Our study suggests that a binomial or presence-absence sampling regime is feasible and can be recommended for estimating NFM densities, as we obtained a good fit in the relationship between the percentage of leaves occupied by mites and the mean number of mites per leaf (r^2^ = 0.67, results not shown). Likewise, female density was positively correlated with the density of all the motile forms (larvae + nymphs + females) (r^2^ = 0.88), indicating that the complete population density can be efficiently estimated by counting only females, which are larger and easy to recognize. Finally, we obtained a good fit between the density of all motile forms on the lower surface and the total density (r^2^ = 0.96), suggesting that either enumerative or binomial sampling could be performed examining only the lower surface.

An accurate integrated strategy to keep population densities under control should include the natural enemies. However, during our observations the presence of predatory mites and insects was only occasional. Only a few individuals of the predatory mite species *Euseius stipulatus* (Athias-Henriot), *Typhlodromus phialatus* Athias-Henriot and *Typhlodromus rhenanoides* Athias-Henriot (Mesostigmata: Phytoseiidae) were found on the leaves in association with NFM. Also, immatures of the predatory thrips *Franklinothrips megalops* (Trybom) (Thripidae) were observed directly feeding on eggs and motile forms of NFM. Given the limited number of predators observed and the intense outbreak of the pest in summer, we consider the impact of this mortality factor to be negligible.
